# Genipin increases oxaliplatin-induced cell death through autophagy in gastric cancer

**DOI:** 10.7150/jca.34773

**Published:** 2020-01-01

**Authors:** Bo Ram Kim, Yoon A Jeong, Dae Young Kim, Jung Lim Kim, Soyeon Jeong, Yoo Jin Na, Hye Kyeong Yun, Seong Hye Park, Min Jee Jo, Hassan Ashktorab, Duane T Smoot, Dae-Hee Lee, Sang Cheul Oh

**Affiliations:** 1 Department of Oncology, Korea University Guro Hospital, Korea University College of Medicine, Seoul, Republic of Korea.; 2 Graduate School of Medicine, Korea University College of Medicine, Seoul, Republic of Korea.; 3 Department of Medicine, Howard University, Washington, District of Columbia, 20060, USA.; 4 Department of Medicine, Meharry Medical Center, Nashville, TN, 37208, USA.

**Keywords:** Oxaliplatin, Genipin, p53, Autophagy.

## Abstract

Oxaliplatin is used for treatment in combination with many drugs. However, the survival rate is still low due to side effects and drug resistance. Therefore, the combination with natural products was required for increasing efficacy and reducing side effects.

Genipin, a natural product derived from the *Gardenia jasminoides*, associated with anti-angiogenic, anti-proliferative, hypertension, inflammatory, and the Hedgehog pathway. It is not known that genipin increases the therapeutic effect of oxaliplatin in gastric cancer. In this study, we found that genipin sensitizes oxaliplatin-induced apoptosis for the first time using colony forming assay, FACS analysis, and western blotting in gastric cancer. Additionally, genipin induced p53 expression in AGS, MKN45, and MKN28 cells. Also, genipin induced autophagy and LC3 expression. Knockdown of LC3 decreased cell death enhanced by the combination of oxaliplatin and genipin. In summary, we showed that genipin increases the oxaliplatin-induced cell death via p53-DRAM autophagy. Based on this, we suggest that genipin is a sensitizer of oxaliplatin.

## Introduction

Gastric cancer is frequently occurring disease of cancer-related death worldwide 1. Recently, although the survival rate of gastric cancer patients has increased, it is still low in a 5-year overall survival 2. The combination chemotherapy such as cisplatin/5-FU, docetaxel/cisplatin/5-FU, and epirubicin/cisplatin/5-FU has been considered as a standard treatment of gastric cancer 3. However, these treatments are still insufficient due to side effects, resistance, and low efficacy. The combination therapies with other natural products are needed to enhance chemo-efficacy decrease side effects.

Oxaliplatin, as alternative to cisplatin, is a third generation platinum agent. Oxaliplain is forming platinum-DNA adducts to disrupt DNA transcription and replication, resulting in DNA damage and apoptosis 4, 5. However, because oxaliplatin has side effects, it is necessary to increase anti-cancer drug efficacy and reduce side effects by combining with natural products.

Genipin, a natural product derived from the *Gardenia jasminoides* 6, associated with anti-angiogenic, anti-proliferative, hypertension, inflammatory, and the Hedgehog pathway 7-9. Genipin has anti-cancer effect in various cancers, such as gastric cancer, colorectal cancer, hepatocellular carcinoma 10, 11. It is not known that genipin increases the therapeutic effect of oxaliplatin in gastric cancer.

Autophagy, a conserved degradation process, is associated with catabolic processes, autoimmunity, cellular development, and cell death 12-14. Autophagy is called type II programmed cell death, unlike apoptosis. However, both apoptosis and autophagy can be induced by similar upstream signaling pathways 15. As LC3 is a marker of autophagosomal membranes, change of LC3-II expression are associated with the autophagic activity via lysosome 16.

p53, tumor suppressor gene, is known to regulate the BH3-domain proteins PUMA and NOXA 17. Moreover, p53 has been shown to play a critical function in autophagy 18, 19. Damage-regulated autophagy modulator (DRAM), a phylogenetically ancient lysosomal protein that contributes to p53-induced autophagy 20. Recently, in the previous studies, p53-DRAM induced autophagy has been also shown to enhance cancer therapy by increasing cytotoxicity of breast cancer cells 21.

In this study, we investigated whether genipin sensitizes oxaliplatin-induced apoptosis in gastric cancer. We found that genipin increased p53 expression. Additionally, we found that genipin induced autophagy. In particular, genipin enhanced autophagy through increase of DRAM. Collectively, we suggest that genipin increases the oxaliplatin-induced cell death via p53-DRAM autophagy in gastric cancer.

## Materials and methods

### Cell culture

Human Gastric cancer cell lines, AGS, MKN45, and MKN28 were purchased from the Korea Cell Line Bank. Cells were cultured in RPMI 1640 medium (Invitrogen, Carlsbad, CA, USA) with 10% fetal bovine serum (Sigma-Aldrich, St. Louis, MO, USA), 1 mM L-glutamine, and 26 mM sodium bicarbonate for monolayer cell culture. The gastric epithelial cell line, HFE-145 was gifted from Dr. Hassan Ashktorab (Howard University). All cell lines were grown at 37ºC in a humidified chamber with 5% CO_2_.

### Reagents and antibodies

Genipin was purchased from Calbiochem (San Diego, CA, USA). Oxaliplatin was purchased from Sigma-Aldrich (St. Louis, MO, USA). Anti-Bak, anti-Bcl-2, anti-Mcl-1, anti-Bcl-xL, anti-p53, and anti-DRAM antibodies were purchased from Santa Cruz Biotechnology (Santa Cruz, CA, USA). Anti-XIAP, anti-NOXA, anti-BIM, anti-survivin, anti-cleaved PARP, anti-caspase-3, anti-caspase-9, and anti-LC3 were purchased from Cell Signaling Technology (Danvers, MA, USA). Anti-actin antibody was purchased from Sigma-Aldrich. For the secondary antibodies, anti-mouse IgG HRP and anti-rabbit IgG HRP were purchased from Cell Signaling Technology.

### Western blotting

Cells were lysed in RIPA buffer (50 mM Tris, 150 mM NaCl, 1% Triton X-100, 0.1% SDS, and 1% Na deoxycholate [pH 7.4]) with a protease and phosphatase inhibitor cocktail (Sigma-Aldrich). Protein concentrations were measured using the bicinchoninic acid protein assay reagent (Thermo Fisher Scientific, Waltham, MA, USA). Equal amounts of proteins were separated by SDS-PAGE and transferred to nitrocellulose membranes (GE Healthcare Life Sciences, Little Chalfont, UK). The membranes were blocked with TBS containing 0.2% Tween 20 and 5% skim milk, incubated with primary antibodies overnight at 4ºC, and then incubated with HRP-labeled secondary antibodies. The signals were detected by X-ray film.

### Flow cytometry analysis of cell apoptosis

The translocation of phosphatidylserine, an apoptosis marker, from the inner to the outer leaflet of the plasma membrane was detected by the binding of allophycocyanin-conjugated annexin V. Briefly, HCT116 cells untreated or treated with genipin, oxaliplatin, or a combination of the two agents were resuspended in the binding buffer provided with the Annexin V-FITC Apoptosis Detection Kit (BioBud, Cat. No. LS-02-100). Cells were mixed with 1.25 μL of the Annexin V- 7 μL FITC reagent and incubated for 30 min at 4°C in the dark. The staining was then terminated and cells were immediately analyzed by flow cytometry.

### Small interfering RNA (siRNA)

p53 siRNA, LC3 siRNA, and negative control siRNA were purchased from Santa Cruz Biotechnology. Cells were transfected with siRNA oligonucleotides using Lipofectamine RNAi Max reagents (Invitrogen) according to the manufacturer's instructions.

### Autophagy detection

Cells grown on glass coverslips were treated with genipin for 24h. After 24h, the medium removed and washed the cells twice with 1X assay buffer. Cells treated microscopy dual detection reagent 100 µL for 30 min at 37°C. Cells were washed 1X assay buffer, followed by fixation 4% formaldehyde for 20 min. The nuclei were co-stained with DAPI. The cells were mounted in Vectashield mounting medium (Vector laboratories, Burlingame, CA, USA) and visualized by fluorescence microscopy.

### Immunofluorescence staining

Cells grown on glass coverslips were fixed with 3.7% formaldehyde for 15 min, followed by permeabilization with 0.5% Triton X-100 for 15 min at room temperature. Cells were then blocked for 1 h with 3% bovine serum albumin and probed with primary antibodies overnight at 4°C, followed by incubation with secondary Alexa fluor-594-conjugated secondary antibody (Molecular Probes, Eugene, OR, USA) or FITC-conjugated secondary antibody (Sigma-Aldrich). The nuclei were co-stained with DAPI. The cells were mounted in Vectashield mounting medium (Vector laboratories, Burlingame, CA, USA) and visualized by fluorescence microscopy.

### Combination Index and Statistical analysis

To determine whether the cytotoxic interactions of genipin and oxaliplatin were synergistic, additive, or antagonistic in gastric cancer cells, drug effects were examined using the combination index (CI) method of Chou and Talalay. GraphPad InStat 6 software was used for all statistical analyses (GraphPad Software, Inc., La Jolla, CA, USA). Statistics were analyzed by using one-way ANOVA with GraphPad InStat 6. One-way ANOVA followed by Turkey post hoc tests were performed in all statistical analysis. To determine the significance between two groups, an unpaired *t* test was used, where a *p*-value of less than 0.05 was considered significant.

## Results

### Genipin enhances oxaliplatin-induced cell death in gastric cancer cell lines

**Fig 1:**
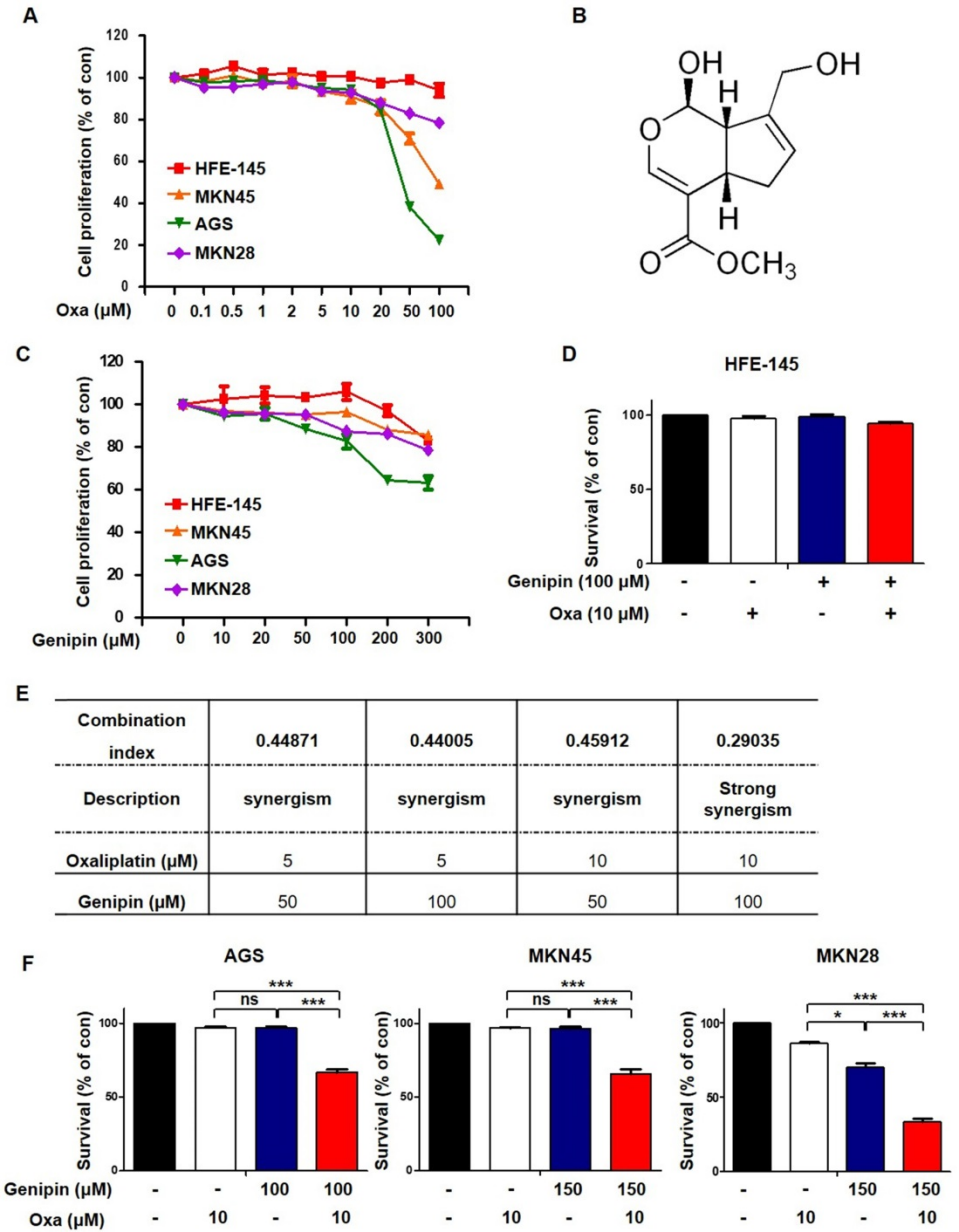
** Genipin enhances oxaliplatin-induced cell death in gastric cancer cell lines.** (A) Treatment with 0-100 µM oxaliplatin for 24 h in gastric cancer cell lines and HFE-145. (B) Molecular structure of genipin. (C) Cell proliferation of gastric cancer cell lines was measured by using the MTT assay after treatment with 0-300 µM genipin for 24h (D) Cell survival of gastric epithelial cell line HFE-145 was measured using trypan blue staining. (E) Combination index for combination of genipin and oxaliplatin, CI = 0.1-0.3, Strong Synergism; CI= 0.3-0.7, Synergism. (F) Cell survival of gastric cancer cells were measured using trypan blue staining. The data are expressed as the mean of three independent experiments. ***P < 0.001, *P < 0.05, ns : not significant.

We first performed an MTT assay to detect cell death induced by oxaliplatin. Oxaliplatin increased cell death in gastric cancer cells (Fig. 1A). To investigate whether genipin increased oxaliplatin-induced cell death, we performed an MTT assay. Genipin induced cell death in gastric cancer cells (Fig 1B and Fig 1C). We examined a combination index (CI) for selecting the most effective concentration using the Compusyn software. As shown in Fig 1E, the combination of 10 µM oxaliplatin and 100 µM genipin showed the best combination effect (Fig 1E). Next, we examined cell death using trypan blue staining. The combination of oxaliplatin and genipin increased cell death in AGS, MKN45, and MKN28 (Fig 1F), but not in gastric epithelial cell (HFE-145) (Fig 1D). These results indicated that genipin enhanced the sensitivity of oxaliplatin in gastric cancer.

### The combination of oxaliplatin and genipin induces apoptosis

**Fig 2:**
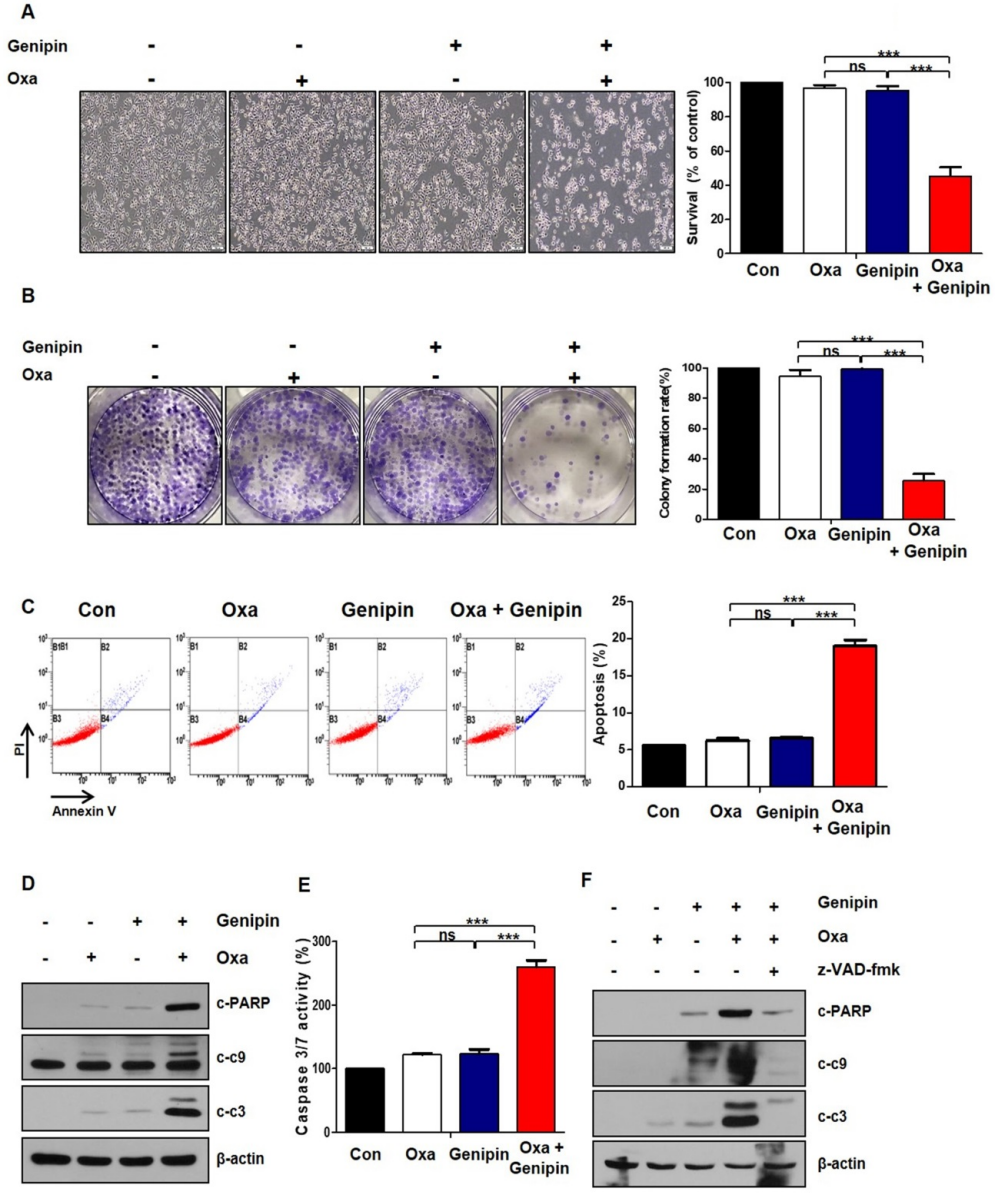
** The combination of oxaliplatin and genipin induces apoptosis.** (A) AGS cells were treated with genipin, oxaliplatin, or combination for 24h. The cells were observed by light microscopy. Scale bar: 100 μm. (B) AGS cells were treated with genipin, oxaliplatin, or combination for 24h. After 10 days, the cells were stained with crystal violet dye, and photographs of the colonies were obtained. (C) AGS cells were treated with genipin, oxaliplatin, or combination for 24h. The cells were stained with annexin V and PI and then were measured using FACS analysis. (D) The activity of cleaved-PARP and cleaved-caspase 3, and cleaved-caspase 9 were measured by western blotting. β-Actin was used as a loading control for each lane. (E) AGS cells were treated with genipin, oxaliplatin, or combination for 24h. Caspase 3/7 activity was measured. (F) AGS cells were pretreated with with 25 µM z-VAD-fmk for 30 min and then treated with oxaliplatin, genipin, or combination. The activity of cleaved-PARP and cleaved-caspase 3, and cleaved-caspase 9 were measured by western blotting. β-Actin was used as a loading control for each lane. The data are expressed as the mean of three independent experiments. ***P < 0.001, ns : not significant.

The combination effects of oxaliplatin and genipin on AGS cells morphology was observed under a light microscope (Fig 2A). Colony forming ability was decreased in the combination of oxaliplatin and genipin, compared to oxaliplatin or genipin alone (Fig 2B). To determine whether the effect of combination induced apoptosis, we performed Annexin V/ PI staining with FACS analysis. The combination of oxaliplatin and genipin increased significantly apoptosis (Fig 2C). Additionally, we examined the activation of cleaved poly (ADP-ribose) polymerase (PARP), caspase-9, and caspase-3. The combination of oxaliplatin and genipin increased the cleaved forms of these proteins (Fig 2D). The activity of caspase 3/7 was increased in the combination of oxaliplatin and genipin (Fig 2E). To investigate whether the combination effects was depended on caspase, we pretrested z-VAD-fmk, a pan-caspase inhibitor. As expected, z-VAD-fmk inhibited the cleaved forms of PARP, caspase-9, and caspase-3 induced by the combination (Fig 2F).

### Genipin enhances oxaliplatin-induced cell death via upregulating p53

**Fig 3:**
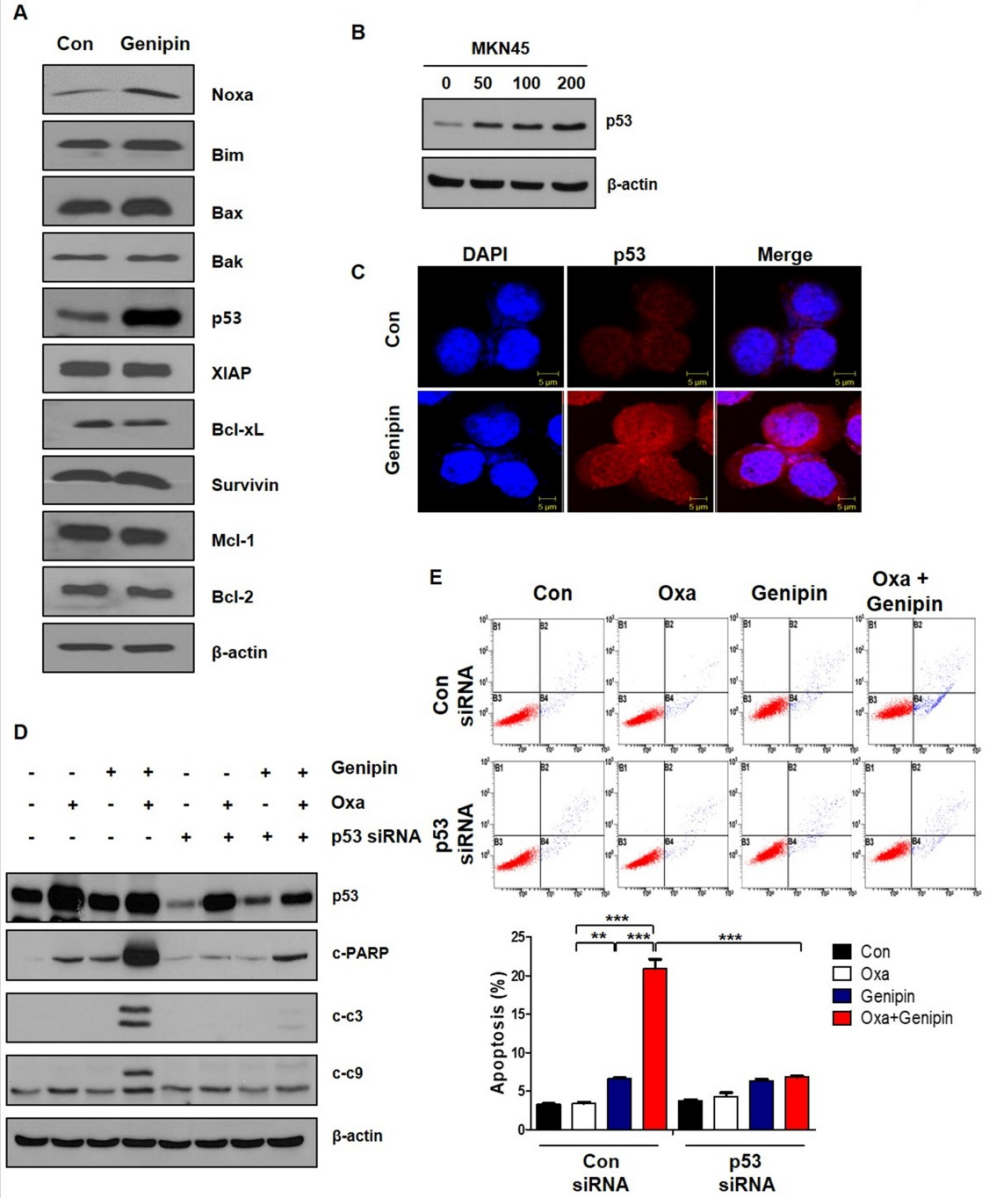
** Genipin enhances oxaliplatin-induced cell death via upregulating p53.** (A) AGS cells were treated with genipin 100 µM for 24h. The apoptotic proteins and anti-apoptotic proteins were measured by western blotting. β-Actin was used as a loading control for each lane. (B) MKN45 cells were treated genipin for 24h. The protein expression of p53 were measured by western blotting. (C) Immunofluorescence of p53 was detected by confocal laser-scanning microscopy (original magnification: 40×). Scale bar: 5 µM (D) AGS cells were transfected with control siRNA or p53 siRNA and then the cells were treated with oxaliplatin, genipin, or combination. The activity of cleaved-PARP and cleaved-caspase 3, and cleaved-caspase 9 were measured by western blotting. (E) AGS cells were transfected with control siRNA or p53 siRNA and then the cells were treated with oxaliplatin, genipin, or combination. The cells were stained with annexin V and PI and then were measured using FACS analysis. ***P < 0.001, **P < 0.01.

To determine how genipin increased sensitivity of oxaliplatin, we confirmed pro-apoptotic proteins and anti-apoptotic proteins. As shown in Fig 3A, genipin increased expression of p53. Upregulation of p53 by genipin was also confirmed in MKN45 cells (Fig 3B). Additionally, increase of p53 by genipin was confirmed by immunofluorescence (Fig 3C). To verify whether upregulation of p53 by genipin enhanced sensitivity of oxaliplatin, we inhibited p53 using p53 siRNA. We found that the combination effect was decreased by p53 siRNA (Fig 3D). Knockdown of p53 significantly decreased apoptosis using FACS analysis (Fig 3E). These results suggest that genipin enhanced oxaliplain-induced apoptosis through upregulation of p53.

### Genipin increases oxaliplatin-induced cell death via autophagy

**Fig 4:**
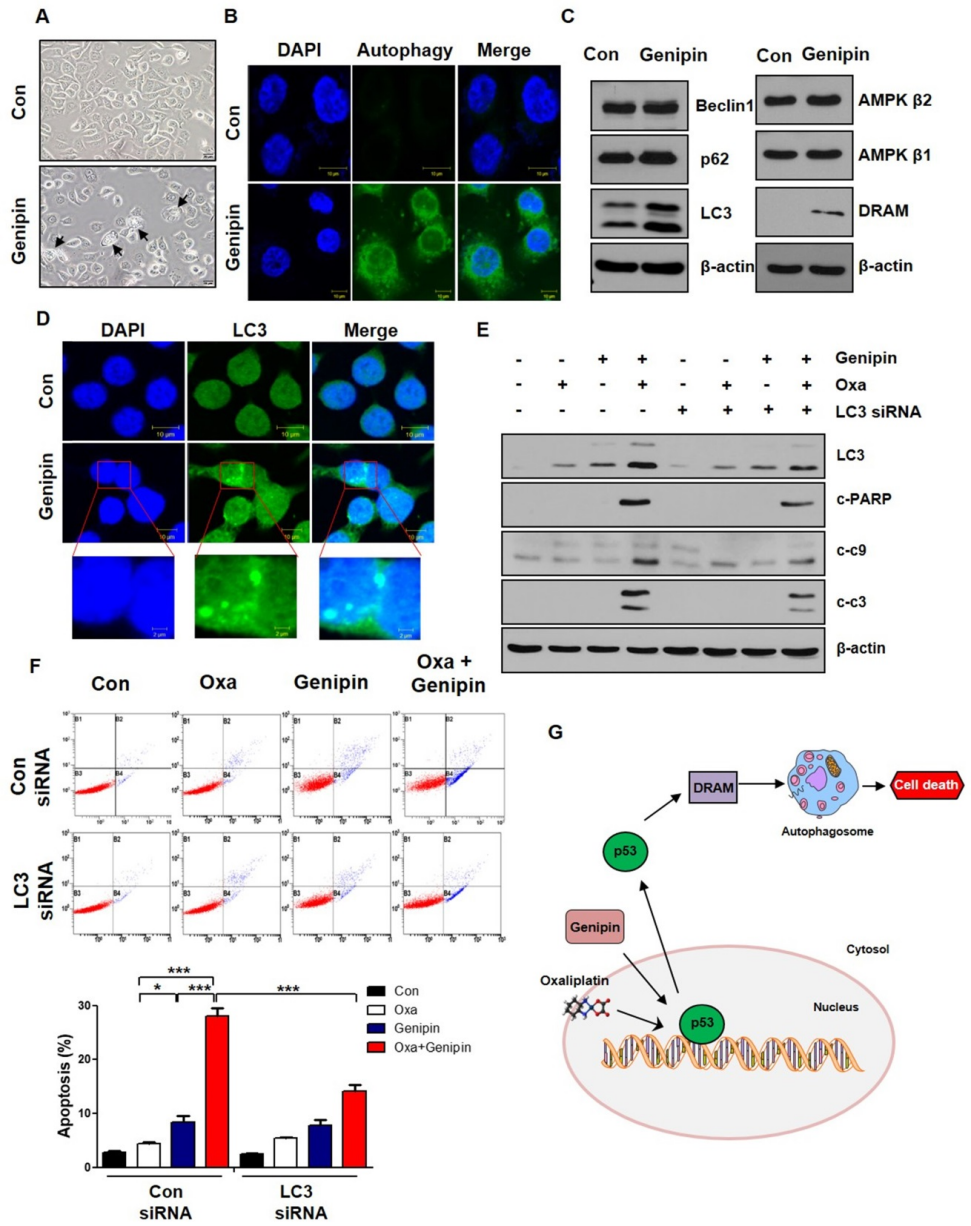
** Genipin increases oxaliplatin-induced cell death via autophagy.** (A) AGS cells were treated with genipin 100 µM for 24h. The cells were observed by light microscopy. Scale bar: 20 μm. (B) The autophagy was observed by immunofluorescence using autophagy detection kit (original magnification: 40×). Scale bar: 10 µM. (C) AGS cells were treated with genipin 100 µM for 24h. The protein expression of Beclin1, p62, LC3, AMPK β1, AMPK β2, and DRAM were measured by western blotting. β-Actin was used as a loading control for each lane. (D) The LC3 puncta were observed by immunofluorescence (original magnification: 40×). Scale bar: 10 µM. (E) AGS cells were transfected with control siRNA or LC3 siRNA and then the cells were treated with oxaliplatin, genipin, or combination. The activity of cleaved-PARP and cleaved-caspase 3, and cleaved-caspase 9 were measured by western blotting. (F) AGS cells were transfected with control siRNA or LC3 siRNA and then the cells were treated with oxaliplatin, genipin, or combination. The cells were stained with annexin V and PI and then were measured using FACS analysis. (G) Schematic diagram for combination model of oxaliplatin and genipin. ***P < 0.001, *P < 0.05.

As shown in Fig 4A, autophagic morphology was appeared when AGS cells were treated with genipin. To determine whether genipin induced autophagy, we performed autophagy staining. Genipin induced autophagy compared to control (Fig 4B). We also confirmed autophagy-related proteins such as Beclin1, p62, and LC3. As shown in Fig 4C, genipin increased LC3 expression. Additionally, genipin increased DRAM. These indicated that genipin induced autophagy via p53-DRAM pathway. Consistent with protein level, genipin increased LC3 puncta using immunofluorescence (Fig 4D). To further confirm whether the combination effect of oxaliplatin and genipin is LC3-dependent, we silenced LC3 using LC3 siRNA. LC3 knockdown decreased cell death induced by the combination of oxaliplatin and genipin (Fig 4E). Additionally, LC3 knockdown significantly decreased apoptosis by FACS analysis (Fig 4F). These results suggest that genipin increases sensitivity of oxaliplatin by inducing autophagy (p53-DRAM).

## Discussion

Oxaliplatin is widely used by combination with other drugs such as 5-FU or folinic acid. However, drug resistance and side effects is still a problem. For this problem, we must overcome these by combination with natural products that can increase the effect and reduce side effects. We found that sensitivity of oxaliplatin was increased through the combination with genipin for the first time in gastric cancer.

Our previous study, we found that genipin enhanced oxaliplatin-induced apoptosis in colorectal cancer 22. In our study, we investigated whether genipin enhanced oxaliplatin induced cell death for gastric cancer. As shown in Fig 1, the combination of oxaliplatin and genipin increased cell death in AGS, MKN45, and MKN28. Additionally, the effect of combination these was confirmed using colony-forming assay, FACS analysis, and western blotting (Fig 2). Our results also showed that p53 is important factor for oxaliplatin sensitivity. Knockdown of p53 decreased genipin-induced oxaliplatin cell death (Fig 3D and Fig 3E).

Autophagy is closely related to cell survival pathway in eukaryotes. It associated with the degradation of cellular components such as long-lived proteins, damaged organelles, protein aggregates, and intracellular pathogens 23. As shown in Fig 4A, we observed autophagic morphology. We also confirmed autophagy induction using autophagy detection kit (Fig 4B). Because genipin increased p53 expression, we confirmed autophagy factors associated with p53 pathway. Genipin significantly increased DRAM expression (Fig 4C). In the previous studies, cytoplasmic p53 is known to suppress autophagy through the activation of mTOR signaling and the inactivation of AMP kinase, whereas nuclear p53 activates autophagy by activation of DRAM which enhances the formation of autophagolysosomes 20, 24. Knockdown of LC3 decreased genipin-induced oxaliplatin cell death (Fig 4E and Fig 4F).

The connection between autophagy and apoptosis is still controversial. It is not yet clear whether autophagy inhibits apoptosis or whether autophagy activates apoptosis, but both cause cell death through by similar upstream signaling pathways 15. In this study, we suggest that genipin enhances oxaliplatin-induced cell death via p53-apoptosis pathway and p53-DRAM-autophagy pathway (Fig 4G).

Recently, combination therapies with more than 30 natural products have been undergo in clinical trials for cancer treatment, but studies on the combined effects of genipin and oxaliplatin are also needed in gastric cancer patients-derived cells.

Taken together, our study can be the baseline research for future clinical trials and suggests genipin as a novel sensitiser of oxaliplatin.

## Abbreviations

PARP: poly (ADP-ribose) polymerase
PBS: phosphate-buffered saline solution
SDS-PAGE: sodium dodecyl sulfate polyacrylamide gel electrophoresis
PMSF: phenylmethylsulfonyl fluoride
TBS: Tris-buffered saline
DAPI: 4,6-diamidino-2-phenylindole
siRNA: short interfering RNA
HRP: horseradish peroxidase
RIPA: radioimmunoprecipitation assay
TBS: Tris-buffered saline
FITC: fluorescein isothiocyanate
PMSF: phenylmethylsulfonyl fluoride
